# Neuromagnetic brain activities associated with perceptual categorization and sound-content incongruency: a comparison between monosyllabic words and pitch names

**DOI:** 10.3389/fnhum.2015.00455

**Published:** 2015-08-17

**Authors:** Chen-Gia Tsai, Chien-Chung Chen, Ya-Chien Wen, Tai-Li Chou

**Affiliations:** ^1^Graduate Institute of Musicology, National Taiwan UniversityTaipei, Taiwan; ^2^Neurobiology and Cognitive Science Center, National Taiwan UniversityTaipei, Taiwan; ^3^Department of Psychology, National Taiwan UniversityTaipei, Taiwan; ^4^Graduate Institute of Brain and Mind Sciences, National Taiwan UniversityTaipei, Taiwan; ^5^Graduate Institute of Linguistics, National Taiwan UniversityTaipei, Taiwan

**Keywords:** pitch name, speech, categorization, semantic, MEG

## Abstract

In human cultures, the perceptual categorization of musical pitches relies on pitch-naming systems. A sung pitch name concurrently holds the information of fundamental frequency and pitch name. These two aspects may be either congruent or incongruent with regard to pitch categorization. The present study aimed to compare the neuromagnetic responses to musical and verbal stimuli for congruency judgments, for example a congruent pair for the pitch C4 sung with the pitch name *do* in a C-major context (the pitch-semantic task) or for the meaning of a word to match the speaker’s identity (the voice-semantic task). Both the behavioral data and neuromagnetic data showed that congruency detection of the speaker’s identity and word meaning was slower than that of the pitch and pitch name. Congruency effects of musical stimuli revealed that pitch categorization and semantic processing of pitch information were associated with P2m and N400m, respectively. For verbal stimuli, P2m and N400m did not show any congruency effect. In both the pitch-semantic task and the voice-semantic task, we found that incongruent stimuli evoked stronger slow waves with the latency of 500–600 ms than congruent stimuli. These findings shed new light on the neural mechanisms underlying pitch-naming processes.

## Introduction

The relationships between language and music, as well as their origins, have been the subject of intensive multidisciplinary research. There are studies comparing the neural substrates underlying the processing of music’s and language’s acoustic/structural features (Zatorre et al., 2002; Wong et al., 2008; Rogalsky et al., 2011), meanings (Koelsch et al., 2004; Steinbeis and Koelsch, 2008, 2011; Daltrozzo and Schön, 2009), combination rules (Patel, 2003; Koelsch et al., 2005; Maidhof and Koelsch, 2011), and motor expressions (Ozdemir et al., 2006; Hickok et al., 2009; Wan et al., 2011; Tsai et al., 2012). Among these aforementioned processes, the perceptual organizations of music and speech are little known in terms of their neural substrates for music and language. Like language, perceptual categorization of musical pitches also relies on symbols in numerous human cultures. Unlike natural sounds or spoken sounds in which fundamental frequencies are continuously distributed, musical pitches are often categorized into discrete entities and are given labels. For example, the pitch names of the major mode scale in Western music are: *do*, *re*, *mi*, *fa*, *sol*, *la*, and *ti*. The current study aimed to examine the perceptual organizations of music (i.e., pitch) and speech (i.e., speaker identity).

### Perceptual Organization of Music: Pitch

According to the mapping rules for associative transformation from a perceived frequency to a pitch name, systems of pitch naming (solmization) can be divided into two types, as represented by the fixed-*do* solmization and the moving-*do* solmization in Western music. The pitch names in the fixed-*do* solmization are determined by the fundamental frequency of auditory stimuli. On the other hand, the moving-*do* solmization relies on pitch relationships and is associated with the use of musical scales. The notes of a scale tend to be arranged unevenly within the octave, with some pitch steps bigger than others (Ball, 2008; Honingh and Bod, 2011). Different musical scales are characterized by different arrangements of pitch steps, namely, by different divisions of an octave. Each note in a scale has unique pitch relationships to other notes and these relationships allow a listener with good relative pitch to label perceived pitches using the moving-*do* solmization. Relative pitch relies on pitch relationships to label pitches. An interesting finding was that the solmization strategy in possessors of absolute pitch differs from that in possessors of relative pitch. Absolute pitch is a rare ability to identify a musical pitch without the use of an external reference pitch. On the other hand, relative pitch possessors identify a musical pitch with the use of an external reference pitch and/or a tonal context. Relative pitch possessors use the moving-*do* solmization, while absolute pitch possessors tend to use the fixed-do solmization (Miyazaki, 2000).

A sung pitch name informs us the fundamental frequency and pitch name that may be either congruent or incongruent with regard to pitch categorization, and a few experiments have used Stroop-like paradigms to study the congruency effect of pitch and pitch name (Itoh et al., 2005; Akiva-Kabiri and Henik, 2012; Schulze et al., 2013). With an auditory Stroop task, Miyazaki (2000) found that relative pitch possessors and absolute pitch possessors tended to use the moving-*do* solmization and the fixed-do solmization, respectively. Schulze et al. (2013) recruited musicians with absolute pitch and musicians with relative pitch (and without absolute pitch) to examine the neural substrates underlying solmization using functional magnetic resonance imaging (fMRI). They used tonal sequences as stimuli with half of these sequences being congruent (e.g., the pitch of C sung as *do*) and half incongruent (e.g., the pitch of C sung as *fa*). Their results showed that detecting verbal-tonal incongruencies activated the left superior temporal gyrus/sulcus (STG/STS) in absolute pitch possessors but not in relative pitch possessors, suggesting the involvement of semantic processing in conflict-monitoring for pitch-naming. Using the event-related potentials (ERP) technique and a Stroop-like paradigm, Itoh et al. (2005) showed that sung pitch names at incongruent pitches evoked stronger positive slow waves 450–550 ms after the stimulus onset compared to congruent stimuli. They also found that absolute pitch possessors elicited a component 150 ms after the stimulus onset in both passive listening and pitch-naming conditions. This suggests the involvement of automatic semantic processing in absolute pitch possessors during music listening. This result is in line with a behavioral experiment of the Stroop effect by Akiva-Kabiri and Henik (2012), which reported that only absolute pitch possessors were unable to ignore the auditory tone when asked to read the note. These studies support the long-held belief that pitch identification in absolute pitch possessors is automatic and impossible to suppress. The present study focused on relative pitch, which is a common ability and its neural correlates are still not fully understood.

### Perceptual Organization of Speech: Speaker Identity

In the language domain, the Stroop color-word test has been widely used to study the conflict processing in the visual modality, whereas the experimental data for spoken words is relatively scant. Haupt et al. (2009) explored the neural underpinning of an auditory Stroop task using fMRI. Their participants were presented with the words “high” or “low” in either high- or low-pitched voice, focusing either on tone pitch (relatively high or low) or on the meaning of a spoken word (high/low/good). The results showed greater activation in the anterior cingulate cortex and the pre-supplementary motor area due to task-related and sensory-level interference. Henkin et al. (2010) investigated the auditory conflict processing using ERP and behavioral measures during Stroop tasks. Their participants were asked to classify word meaning or speaker’s gender while ignoring the irrelevant speaker’s gender or word meaning, respectively. The results showed significantly reduced N1 amplitude and prolonged N4 in the speaker’s gender task compared to the word meaning task. Using a similar auditory Stroop test, Christensen et al. (2011) demonstrated a significant interference effect with gender-typical nouns spoken by gender-mismatched voices, and the fMRI data showed that interference-related activation was localized ventrally in medial frontal areas. The methodology of these studies provides evidence of exploring the neural correlates of the perceptual organizations of speech.

### Comparison of Music and Speech: Sound-Content Incongruency

While auditory Stroop-like tasks have been studied in the domains of music and speech, no experiment has directly compared the congruency effect that these two domains have on brain activities. This may be due to the distinct acoustical features of music and speech. All pitch names are monosyllabic and the processing units of Chinese words are also monosyllabic (i.e., characters; Tsang and Chen, 2009). The seven notes in Chinese traditional music are represented by monosyllabic Chinese words (*shang*, *che*, *gong*, *fan*, *liu*, *wu*, and *yi*). In the present study, we benefited from this characteristic of Chinese language to directly compare the congruency effect of monosyllabic spoken words with that of monosyllabic pitch names. The specific aim of this study was two-fold: to compare the neuromagnetic activities associated with stimulus categorization of sung pitch names and spoken words, and to compare the neuromagnetic activities associated with the detection of sound-content incongruency of these stimuli. To achieve these goals, we presented participants with stimuli conveying two kinds of information for the same concept. A sung pitch name conveys: (1) the acoustic information of pitch in terms of fundamental frequency; and (2) the semantic information of pitch in terms of the pitch name. A spoken word conveys: (1) the phonetic cues of speaker’s gender and age; and (2) the semantic information of gender and age. These auditory stimuli provide a novel opportunity to identify the common and distinct properties of the neural mechanisms underlying music and speech processing. We used magnetoencephalography (MEG) with a high temporal resolution to disentangle information processing stages of musical stimuli and verbal stimuli.

Regarding the hypotheses of the present study, previous electroencephalography (EEG) studies suggest that early processing of perceptual categorization enhances the amplitude of the P2 response around 200 ms after stimulus presentation (Cranford et al., 2004; Tong and Melara, 2007; Tong et al., 2009; Ross et al., 2013). We thus predicted that its neuromagnetic counterpart, P2m, is enhanced in response to musical stimuli of pitch congruent with pitch name compared to incongruent musical stimuli, because the congruent stimuli are associated with a rapid process of pitch categorization. The sources of P2m have been found to be localized in the auditory association cortices (Shahin et al., 2003; Kuriki et al., 2006; Thaerig et al., 2008; Tong et al., 2009; Liebenthal et al., 2010; Ross et al., 2013), which were defined as the regions of interests (ROIs) in this study.

Moreover, we expected the amplitude of late neuromagnetic components to be modulated by semantic conflict during both the music task and the speech task (Christensen et al., 2011). Congruency manipulation of our stimuli allows us to test the semantic N400 effect, which manifests in a larger negative potential with a latency of approximately 400 ms for words that are semantically incongruent to a given context compared to words that are congruent (for a recent review, see Kutas and Federmeier, 2011). We predicted that both musical and verbal stimuli show this N400 effect. Moreover, the conflict processing for incongruent stimuli may elicit late slow waves of magnetic fields (SWm) 500 ms after stimulus onset, as suggested by previous studies on musical pitch (Itoh et al., 2005; Elmer et al., 2013), sentence perception (van Herten et al., 2006; Frenzel et al., 2011), and color-naming Stroop tasks (Larson et al., 2009; Coderre et al., 2011).

## Materials and Methods

### Participants

Nineteen volunteers (20–28 years old, 11 females) were recruited by means of a public announcement, which stated the requirement of relative pitch capacity for participation in this study. All of the participants had taken musical lessons for more than 5 years, but none were professional musicians. All of them were right-handed and had normal hearing. The informed consent procedures were approved by the Institutional Review Board of Academia Sinica. Participants gave informed written consent and received monetary compensation for participating in the study. Three participants (two females and one male) were excluded from the data collection because of severe artifactual activity that could be noted in the MEG sensor array. One female participant was excluded on the basis of her low accuracy (<95%) of the pitch-semantic task in the pre-scan session (see Procedure). The data collected from the remaining fifteen participants with good relative pitch were used for the final analysis.

### Stimuli

There were two tasks in this study, the pitch-semantic task and the voice-semantic task. In the pitch-semantic task, the stimuli were pitch names of *do*, *re*, *mi*, and *sol* sung by a semi-professional soprano. The sung pitches of these pitch names were restricted in four notes: C4, D4, E4, and G4. When a pitch matched the pitch name, it was a congruent stimulus in a C-major context, otherwise it was an incongruent stimulus. For example, the pitch C4 sung with the pitch name *do* was a congruent stimulus in a C-major context, whereas this pitch C4 sung with the pitch name *re* was an incongruent stimulus in a C-major context. We did not use the pitch F4 as stimulus because the E4-F4 interval is the minor second (semitone) which is not as large as the intervals between other pitches, and to discern between E4 and F4 might be particularly difficult. Table 1 displays all the combinations of pitch and pitch names used as stimuli in this study. The fundamental frequency of these sung pitch names ranges from 261 to 392 Hz.

**Table 1:** **The sixteen stimuli of the pitch-semantic task**.

Syllables	Pitch names			
Pitch	Congruent	Incongruent		
C4	Do	Re	Mi	Sol
D4	Re	Do	Mi	Sol
E4	Mi	Do	Re	Sol
G4	Sol	Do	Re	Mi

*In congruent stimuli, the pitch and pitch name were matched in a C-major context*.

In the voice-semantic task, we used monosyllabic Chinese words, including: /Nan2/ (male), /Nü3/ (female), /Xiao3/ (child), and /Lau3/ (elder) spoken by a man, a woman, an 8-year-old girl, and a hoarse voice that exaggerated the voice characteristic of elderly men. If the meaning of a word matched the speaker’s identity revealed by the acoustic characteristics of the voice, it was a congruent stimulus, otherwise it was an incongruent stimulus. For example, a word /Nü3/ (female) spoken by a woman was a congruent stimulus, whereas the words “male” or “elder” spoken by a woman were incongruent stimuli. The voices of these four speakers differed in acoustical features. The fundamental frequency of man’s voice was within the range of 120–200 Hz, whereas the fundamental frequency of woman’s and girl’s voices was in the range of 250–400 Hz, with different formant frequencies to differentiate between a woman and a girl (Peterson and Barney, 1952; Fitch, 1997). Finally, an amateur actor mimicked the voice of an elderly man by highlighting the hoarse quality characteristic of vocal fold bowing, which is commonly found in the larynx of elderly men (Pontes et al., 2005). To facilitate the detection of sound-content incongruency, we excluded the male-elder and female-child combinations, because their voices share some acoustic characteristics, and to discern between them might be particularly difficult. Table 2 displays all the combinations of speakers and words used as stimuli in this study.

**Table 2:** **The twelve stimuli of the voice-semantic task**.

Syllables	Words		
Speaker	Congruent	Incongruent	
Adult man	男 /Nan2/ (man)	女 /N ü3/ (woman)	小 /Xiao3/ (young)
Adult woman	女 /N ü3/ (woman)	男 /Nan2/ (man)	老 /Lau3/ (elder)
Male elder	老 /Lau3/ (elder)	女 /N ü3/ (woman)	小 /Xiao3/ (young)
Girl	小 /Xiao3/ (young)	男 /Nan2/ (man)	老 /Lau3/ (elder)

All stimuli were edited and digitized (44,100 Hz sampling rate, 16 bit mono) for presentation using Gold Wave Digital Audio Editor (Gold Wave Inc.). The sound level of the presentation was approximately 60 dB. The duration of these stimuli ranged from 450 to 500 ms. All the musical stimuli had a stable pitch, whereas all verbal stimuli had a gliding pitch.

### Procedure

There were three sessions in this experiment. In the first session, a singing test used a newly-composed melody (B♭, 4 bars) to ensure that participants had a good capacity for relative pitch. They were asked to sing this melody in movable-*do* solmization after hearing it. The participants were admitted to the next stage only if they could sing fluently. All participants passed this singing test.

In the second session, participants listened to the stimuli and practiced using the device for responding. They were instructed to make a button-press response indicating whether the stimulus was sound-content congruent or not (a right button for “congruent” and a left button for “incongruent”). There were two runs (pitch-semantic task and voice-semantic task) in this session, each run comprising 20 trials. Participants would enter the third session (MEG scan) only if their mean response accuracy was higher than 95%. One participant was excluded from the third session as a result of this criterion.

Prior to the MEG data acquisition, each participant’s head shape was digitized, and head position indicator coils were used to localize the position of the participant’s head inside the MEG helmet. The MEG scan consisted of four runs, each lasting approximately 4.5 min. There were 1-min breaks interspersed between these runs, and the total duration of the MEG experiment was approximately 21 min. The tasks in the four runs were: voice-semantic, pitch-semantic, voice-semantic, and pitch-semantic. Because our participants relied on relative pitch, we presented an upward scale and a tonic chord of C major immediately before the two runs for the pitch-semantic task to help participants establish a tonal schema (tonality), which determines the rules of the pitch-to-pitch-name associative transformations.

Each run consisted of 180 trials, including 80% sound-content congruent stimuli and 20% sound-content incongruent stimuli (see Tables 1, 2). This proportion of incongruent stimuli was chosen for maintaining a relatively stable tonal schema. These trials were presented in a pseudorandom order within each run. Each trial lasted 1.5 s, consisting of a sound (a sung pitch name or a spoken word) with the duration of 500 ms, followed by an interstimulus silence. Sounds were delivered binaurally through silicon tubes. Participants were instructed to indicate the sound-content congruency of the stimulus with a button press (right for “congruent” and left for “incongruent”) as quickly as possible.

### Data Acquisition and Analysis

E-Prime was used to present all stimuli and to collect the behavioral data of button-press responses. A two task by two congruency ANOVA with repeated measures was conducted to analyze the reaction time data for correct responses. Based on previous studies (Haupt et al., 2009; Christensen et al., 2011), reaction times exceeding 1000 ms were classified as outliers and did not enter the statistical analysis.

Neuromagnetic brain activities evoked by auditory stimuli were acquired using a 156-channel axial gradiometer whole-head MEG system (Kanazawa Institute of Technology, Kanazawa, Japan) at a sampling frequency of 1 kHz. A band-pass filter (DC to 100 Hz) was applied during the recording. MEG data were processed by a MEG laboratory 2.004A (Yokogawa Electric Corporation). The trials in which participants failed to respond or made incorrect responses were rejected from further analysis. MEG data were first noise reduced, and then epoched with 100 ms prestimulus intervals as well as 800 ms post-stimulus intervals. Trials with amplitude variations larger than 1.5 pT were excluded from further processing. Both the congruent stimuli and incongruent stimuli contained at least 72 artifact-free trials for each participant and each task. These trials were baseline-corrected using the prestimulus data. Each participant’s MEG data within the epoch was averaged across the trials of the same condition, and was low-pass filtered at 30 Hz.

**Figure 1:**
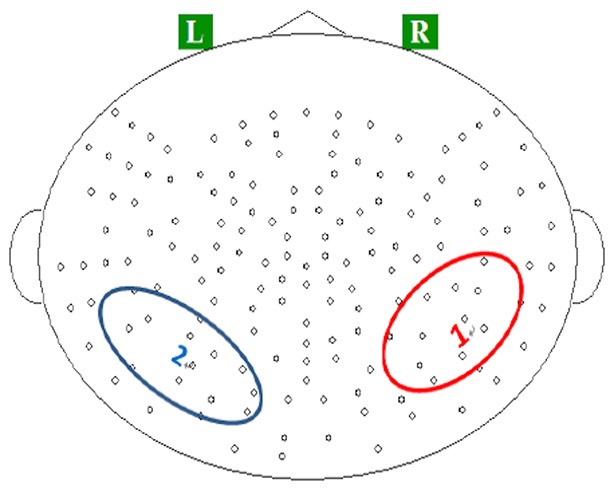
**Locations of regions of interests (ROIs) on (1) the right temporoparietal region, and (2) left temporoparietal region**.

On the basis of previous studies (Itoh et al., 2005; Steinbeis and Koelsch, 2008), we focused on three components of the evoked magnetic fields: P2m, N400m, and SWm. Neuromagnetic responses recorded in temporoparietal channels were evaluated using an ROI analysis. These channels were selected due to their role in auditory categorization based on previous studies (Shahin et al., 2003; Kuriki et al., 2006; Thaerig et al., 2008; Tong et al., 2009; Liebenthal et al., 2010; Rueschemeyer et al., 2014). The ROIs were defined as the pronounced evoked fields in the average topographies for P2m (Figure 1). For each participant, we selected the three channels from the ROIs that recorded strongest P2m. Time courses of the MEG signal in these spatial ROIs were obtained by averaging the waveforms of the event-related field within the epoch of 0–600 ms over these three channels.

Appropriate time windows of measurement were determined by visual inspection of grand averages and individual participant data. A P2m within the 200–230 ms window, an N400m within the 330–430 ms window, and an SWm within the 500–600 ms window. We did not analyze N1m responses because it may be affected by acoustic features such as pitch glides (Mäkelä et al., 2004). The amplitudes of P2m, N400m, and SWm were estimated by averaging the field amplitude over these windows. The amplitude of each component was subjected to a repeated 2 (task) × 2 (stimulus congruency) × 2 (brain hemisphericity) ANOVA.

## Results

**Figure 2:**
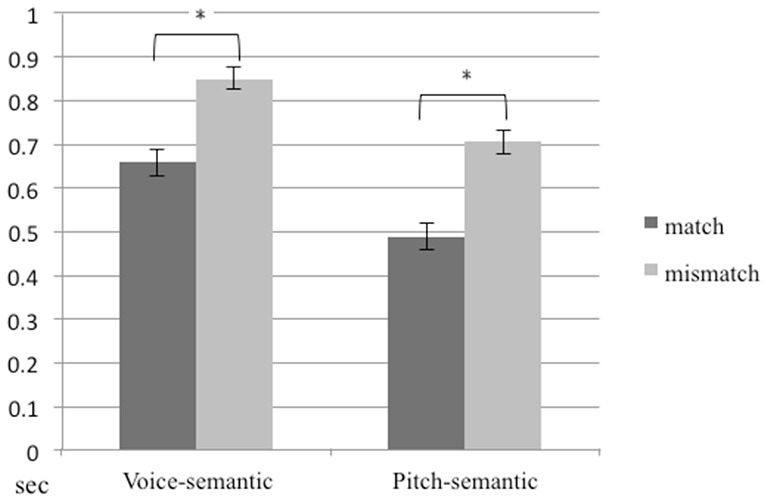
**Reaction times for the pitch-semantic task and the voice-semantic task**. Error bars indicate standard errors of the mean.

The proportional correct for congruency/incongruency detection, averaged across participants, was 97.8%. A 2 (task) × 2 (congruency) repeated ANOVA on reaction time for trials with correct responses showed significant main effects for task (*F*_1,14_ = 85.08, *p* < 0.01) and congruency (*F*_1,14_ = 622.27, *p* < 0.01). The mean of reaction time in the pitch-semantic task was shorter than that in the voice-semantic task. The mean of reaction time for congruent stimuli was shorter than for incongruent stimuli (Figure 2).

**Figure 3:**
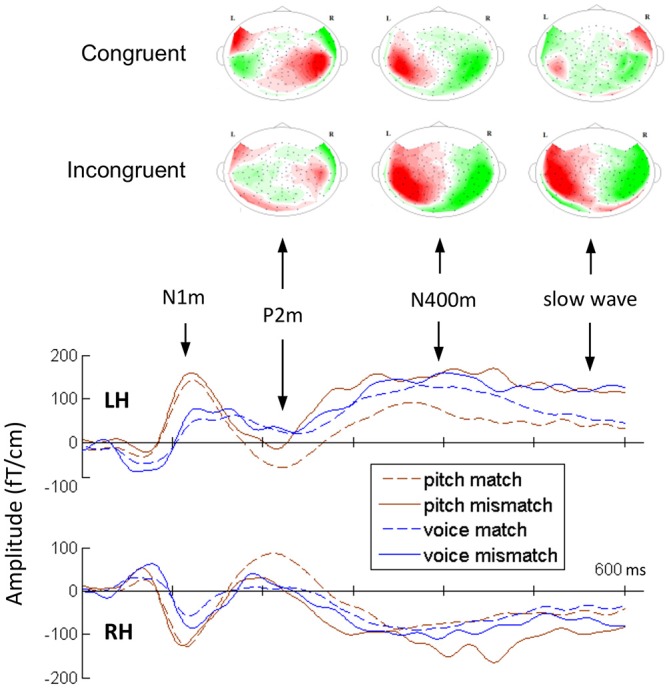
**The average topographies for P2m, N400m, and SWm for two conditions of the pitch-semantic task and the grand-average magnetoencephalography (MEG) waveforms in ROIs**.

**Figure 4:**
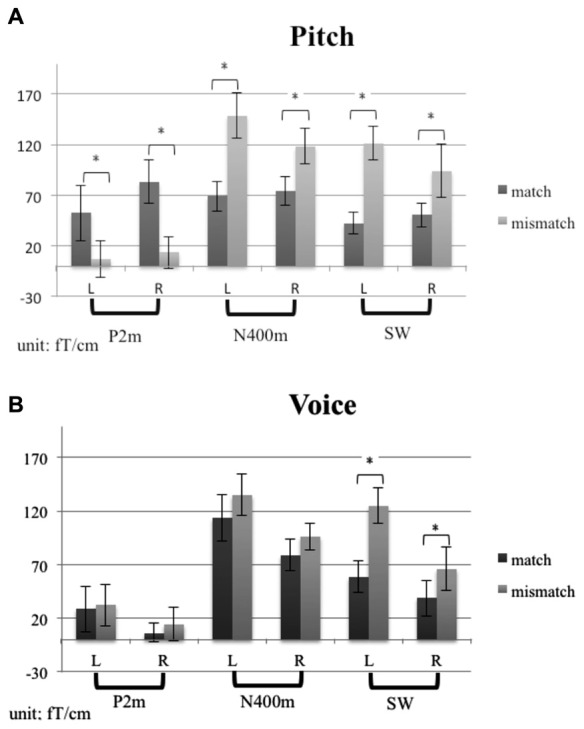
**The mean amplitudes of P2m, N400m, and SWm for (A) the pitch-semantic task and (B) the voice-semantic task**. Error bars indicate standard errors of the mean.

Figure 3 shows the grand average MEG waveform components at 210, 380, and 550 ms for each condition and ROIs. Both the incongruent musical stimuli and incongruent verbal stimuli evoked significant N400m and SWm, whereas congruent stimuli of sung pitch name evoked significant P2m. The mean amplitudes of P2m, N400m, and SWm are presented in Figure 4. The results of ANOVA for peak amplitude are summarized as follows (only significant effects were reported).

*P2m*. A 2 (task) × 2 (congruency) × 2 (hemisphere) repeated ANOVA revealed a main effect of congruency (*F*_1,14_ = 8.33, *p* < 0.05) and a significant interaction between congruency and task (*F*_1,14_ = 4.51, *p* = 0.05). A simple main effects analysis was further conducted, showing that congruent stimuli evoked stronger P2m for the pitch-semantic task compared to incongruent stimuli (*F*_1,28_ = 11.95, *p* < 0.01).

*N400m*. A 2 (task) × 2 (congruency) × 2 (hemisphere) repeated ANOVA revealed a significant main effect of congruency (*F*_1,14_ = 37.26, *p* < 0.01) and an interaction between congruency and task (*F*_1,14_ = 7.40, *p* < 0.05). A simple main effects analysis was further conducted, showing that incongruent stimuli evoked stronger N400m for the pitch-semantic task compared to congruent stimuli (*F*_1,28_ = 36.33, *p* < 0.01).

*SWm*. A 2 (task) × 2 (congruency) × 2 (hemisphere) repeated ANOVA revealed a main effect of congruency (*F*_1,14_ = 38.44, *p* < 0.01). The hemisphere × task × congruency interaction (*F*_1,14_ = 5.3, *p* < 0.05), the hemisphere × task interaction (*F*_1,14_ = 5.05, *p* < 0.05), and task × congruency interaction (*F*_1,14_ = 6.62, *p* < 0.05) were also significant. To further understand the three-way interaction, a 2 (task) by 2 (congruency) repeated ANOVA was calculated for the left hemisphere and the right hemisphere separately. In the left hemisphere, the main effect of congruency was significant (*F*_1,14_ = 21.92, *p* < 0.01). In the right hemisphere, there was a significant interaction effect of task × congruency (*F*_1,14_ = 15.08, *p* < 0.01) and a main effect of congruency (*F*_1,14_ = 34.63, *p* < 0.05). A follow-up simple simple main effects analysis illustrated that incongruent stimuli evoked stronger SWm than congruent stimuli for both tasks (pitch-semantic task, *F*_1,28_ = 49.40, *p* < 0.01; voice-semantic task, *F*_1,28_ = 5.04, *p* < 0.05).

## Discussion

The present MEG study aimed at comparing the neuromagnetic activities associated with perceptual categorization and congruency effects within the music and speech domains. We observed that the detection of semantic congruency/incongruency for musical stimuli occurred earlier than that for verbal stimuli. This pattern emerged in both behavioral data (reaction time) and event-related field components. The reaction time in the pitch-semantic task was significantly shorter than the voice-semantic task, and the reaction time of congruent stimuli was significantly shorter than for incongruent stimuli. Neuromagnetic data suggests that P2m evoked by congruent stimuli of sung pitches was stronger than that evoked by incongruent pitches. In addition, we replicated the previously documented N400 effect of musical stimuli (Steinbeis and Koelsch, 2008, 2011; Daltrozzo and Schön, 2009) but we did not find the N400 effect of verbal stimuli. Furthermore, for both musical and verbal stimuli, SWm evoked by incongruent stimuli was stronger than that evoked by congruent stimuli, as predicted by previous studies on conflict processing (West, 2003; van Herten et al., 2006; Larson et al., 2009; Coderre et al., 2011; Frenzel et al., 2011) and a Stroop-like effect of pitch naming (Itoh et al., 2005).

The earliest neuromagnetic component showing the congruency effect of pitch naming was P2m, which was pronounced 200–230 ms after stimulus onset and enhanced for congruent stimuli. To the best of our knowledge, the present study is the first one reporting that musical pitches elicited a congruency-sensitive component with latency shorter than 230 ms. In the ERP experiment by Itoh et al. (2005), the P2 responses to the musical stimuli with congruent pitch and pitch name appeared stronger than incongruent stimuli when selective attention was focused on pitch (see Figure 5A of their paper). However, they did not perform statistical analysis on the P2 amplitude.

The enhanced P2m response to pitch congruent with pitch name may reflect the activation of short-term memory during a rapid perceptual categorization of auditory stimuli. On the other hand, the incongruent musical stimuli may fail to be classified due to conflicting information, thereby inducing a weaker P2m response. In EEG studies, P2 has been related to the processes of object identification and stimulus classification (Cranford et al., 2004; Tong and Melara, 2007; Tong et al., 2009; Ross et al., 2013). In a combined EEG-fMRI experiment, Liebenthal et al. (2010) compared the pattern of activation in the left STS between familiar phonemic patterns and unfamiliar nonphonemic patterns during categorization. They found stronger P2 responses to phonemic patterns than nonphonemic patterns before training and increased P2 response to nonphonemic patterns with training. The authors argued that P2 may reflect the activation of neural representations of the relevant (trained) sound features providing the basis for perceptual categorization, and the P2 training effect may be related to the activation of new short-term neural representations of novel auditory categories in the left STS (also see Hickok et al., 2003). In this study, participants may have kept short-term memories of sung pitch names in the C-major scale, which served as the tonal schema for pitch categorization. The musical stimuli of pitch congruent with pitch name might activate the short-term neural representations of musical pitch and elicit a prominent P2 response. This view is in line with Marie et al. (2011), who found that metrically incongruous words elicited larger P2 components in musicians compared to metrically congruous words. Their result shows P2 enhancement by a match between the auditory input and the metrical template. Our result also shows the similar effect by a match between the auditory input and the template of pitch names.

Previous findings of music-listening also support the idea that P2 may reflect the activation of neural representations of the trained sound features providing the basis for perceptual categorization. Shahin et al. (2003, 2005) examined whether the auditory-evoked responses were modulated by the spectral complexity of musical sounds, finding larger P2 responses to instrumental sounds in musicians relative to non-musicians. Moreover, the musician’s P2 response to instrumental sounds was enhanced relative to pure tones. Kuriki et al. (2006) also found P2 enhancement for harmonic progressions in musicians. Seppänen et al. (2012) used the oddball paradigm to examine training-induced neural plasticity, finding that P2 amplitude was enhanced after 15 min of passive exposure in both musicians and non-musicians. The P2 training effect was also found in discrimination experiments of amplitude modulated pure tones (Bosnyak et al., 2004) and speech sounds (Tremblay et al., 2009). Our hypothesis of P2 playing a role in categorization is also supported by its topography. Although we did not reconstruct the electromagnetic sources for MEG components, the grand-average topography of P2 shows direction inversion in the magnetic fields around the bilateral superior temporal regions (Figure 3). This provides strong evidence for the existence of current dipoles around these regions. Previous studies have converged to indicate that the sources of the auditory-evoked P2 were located in the mid-posterior regions of STG/STS (Verkindt et al., 1994; Godey et al., 2001; Shahin et al., 2003; Bosnyak et al., 2004; Kuriki et al., 2006; Thaerig et al., 2008; Liebenthal et al., 2010).

Regarding N400m, our finding of an enhanced N400m response to incongruent stimuli for the pitch-semantic task agrees with previous research on the semantic N400 effect, which is reflected in a larger N400 amplitude for words that are semantically incongruent to a given context than words that are congruent. ERP research over recent decades has indicated that N400 can be elicited by a wide range of stimulus types, and its amplitude is sensitive to semantic manipulations (for a recent review, see Kutas and Federmeier, 2011). In the music domain, N400 was mostly examined using an affective priming paradigm. Several studies have revealed that a target musical sound elicits stronger N400 when its prime word (Steinbeis and Koelsch, 2008, 2011; Daltrozzo and Schön, 2009) or facial expression (Kamiyama et al., 2013) has an incongruent emotional meaning. Recently, an N400 semantic priming effect for the congruency of pitch and pitch name was reported to be related to absolute pitch. Elmer et al. (2013) presented musical tones and visual labels of pitches to musicians with/without absolute pitch, finding an increased N400 effect in possessors of absolute pitch, in comparison with non-possessors of absolute pitch. The present study extends previous findings by demonstrating an N400 semantic effect for the concurrent information of acoustic pitch and pitch name in possessors of relative pitch.

To our surprise, we did not find the semantic N400 effect for verbal stimuli. In reality, we seldom feel odd when hearing the word “male” spoken by a female, and* vice versa*. Whereas the detection of the incongruency of the speaker’s identity and word meaning was instructed by the experimenter, detection of the incongruency of pitch and pitch name may be relatively automatic. In the past decade, accumulated evidence suggests that the N400 effects seem to occur implicitly and may be associated with relatively automatic processes (Rolke et al., 2001; Deacon et al., 2004; Kiefer and Brendel, 2006; Kelly et al., 2010; Schendan and Ganis, 2012). In our view, the N400 effect observed in the pitch-semantic task might be attributed to the automaticity of incongruency detection of pitch and pitch name. Previous experiments suggest that N400 amplitude is likely to vary with many of the same factors that influence the reaction time (Gomes et al., 1997; Chwilla and Kolk, 2005; Kutas and Federmeier, 2011; Lehtonen et al., 2012). Moreover, the dipole source of N400 has been consistently suggested to be located in the temporal lobe (Dien et al., 2010; Dobel et al., 2010; Hirschfeld et al., 2011; Kutas and Federmeier, 2011) and is likely associated with sensory and automatic processes.

Given that the mean reaction time in the voice-semantic task was longer than pitch-semantic task for more than 100 ms, the incongruency detection in the voice-semantic task may not be automatic, and therefore this task did not show the N400 effect. It should be noted that the right anterior STG/STS is involved in integrative processing of several acoustical features necessary for speaker identification (Belin and Zatorre, 2003; von Kriegstein et al., 2003; Lattner et al., 2005; Bonte et al., 2014). This may partially explain that the mean reaction time in the pitch-semantic task was shorter than voice-semantic task.

We found that the incongruency detection of the speaker’s identity and mismatched word meaning was significantly slower than the congruency detection of the speaker’s identity and matched word meaning. This suggests the existence of an incongruity and conflict process in the verbal domain. Our view that the voice-semantic task mainly involves controlled processes is supported by a prior study of verbal processing. An interference effect with gender-typical nouns spoken by gender-mismatched voices (e.g., “father” spoken by a woman) was related to a controlled process (Christensen et al., 2011).

As to the final component of SWm (500–600 ms) in both the pitch-semantic task and the voice-semantic task, we found that incongruent stimuli evoked stronger SWm than congruent stimuli. The congruency effect of the voice-semantic task manifests in the left hemisphere, whereas the congruency effect of the pitch-semantic task manifests in both hemispheres. In EPR studies, the late positive slow wave after 500 ms is related to conflict detection and resolution (West, 2003; van Herten et al., 2006; Larson et al., 2009; Coderre et al., 2011; Frenzel et al., 2011). Our result of the congruency effect in the pitch-semantic task is consistent with Itoh et al. (2005), who found enhanced parietal late slow waves for auditory stimuli of pitch incongruent with pitch name in non-possessors of absolute pitch, in comparison to congruent stimuli. In ERP studies, the conflict slow potential reflects greater positivity for incongruent trials than for congruent trials over the parietal region 500 ms after stimulus onset (Liotti et al., 2000; West and Alain, 2000). We hypothesized that SWm may be the magnetic counterpart of the parietal conflict slow potential.

A limitation of this study was that the observed differences in the reaction time and the strength of event-related field components between music and speech may relate to the acoustical properties of these two types of stimuli. While categorization of musical pitch relies on the fundamental frequency of voice, categorization of speaker identity relies on the fundamental frequency and voice quality. The voice quality of speech stimuli varied across speakers, whereas the voice quality of music stimuli did not change. The larger variation of voice quality of speech stimuli may increase the difficulty of the congruency-detection task compared to music stimuli and affects the amplitude of P2m, N400m, and SWm.

The present study benefited from the monosyllabic Chinese words in minimizing the acoustical differences between the speech and music stimuli. While all stimuli were monosyllabic, however, the spoken Chinese words differ from the sung pitch names in the gliding fundamental pitch. Future investigations should assess the effect of the gliding fundamental pitch of lexical tones on semantic processing.

It should be noted that our participants relied on relative pitch, and the enhanced P2m responses to pitch congruent with pitch name reflect the categorization of musical pitches in a given tonal context, which was established by an upward scale and a tonic chord immediately before the two runs for the pitch-semantic task. In contrast, absolute pitch possessors tend to categorize musical pitches with the use of the fixed-do solmization and without the use of an external reference pitch or a tonal context. A detailed comparison between relative pitch and absolute pitch awaits future research.

## Conclusion

We compared the neuromagnetic responses to musical stimuli and verbal stimuli, with the sound-content congruency of these stimuli being manipulated. Detection of the incongruency of the speaker’s identity and word meaning was slower than the detection of the incongruency of pitch and pitch name, as revealed by reaction time and event-related field components. We reported the novel finding of enhanced P2m elicited by pitch congruent with pitch name, which suggests that perceptual categorization of musical pitches occurs earlier than the detection of semantic incongruency reflected by N400m. For verbal stimuli, P2m and N400m did not show any congruency effect. Our results allow for the attribution of the nature and use of musical scales in numerous human cultures. Although the fundamental frequencies of sounds distribute continuously, our cognitive system tends to categorize musical pitches into discrete entities and to label each categorized pitch with a name. We suggest that pitch categorization with the use of the moving-do solmization occurs 200–230 ms after stimulus onset.

## Conflict of Interest Statement

The authors declare that the research was conducted in the absence of any commercial or financial relationships that could be construed as a potential conflict of interest.
